# Engineering Design of an Active–Passive Combined Thermal Control Technology for an Aerial Optoelectronic Platform

**DOI:** 10.3390/s19235241

**Published:** 2019-11-28

**Authors:** Zhifeng Cheng, Lu Sun, Fuhe Liu, Xiaofeng Liu, Lei Li, Quanchao Li, Richa Hu

**Affiliations:** 1 Key Laboratory of Airborne Optical Imaging and Measurement, Changchun Institute of Optics, Fine Mechanics and Physics, Chinese Academy of Sciences, Changchun 130033, China; chengzhifeng@ciomp.ac.cn (Z.C.); liufuhe@ciomp.ac.cn (F.L.); liuxiaofeng@ciomp.ac.cn (X.L.); lilei@ciomp.ac.cn (L.L.); liquanchao@ciomp.ac.cn (Q.L.); huricha@ciomp.ac.cn (R.H.); 2 University of Chinese Academy of Sciences, Beijing 100049, China

**Keywords:** aerial optoelectronic platform, active-passive combined thermal control technology, temperature level, temperature gradient

## Abstract

In order to ensure the imaging performance of the aerial optoelectronic platform system in low temperature environment, an active-passive combined thermal control technology was studied. A thermal control finite element model of the aerial optoelectronic platform was established. Additionally, thermal control simulation analysis and experiments under extreme conditions were carried out respectively. The simulation and experimental results showed that the temperature level of the primary mirror is improved above 25 °C by the proposed thermal control technology effectively, meanwhile the temperature gradient of the primary and secondary mirrors are less than 5 °C. The successful implementation of this active-passive combined thermal control technology provides a technical support for the precision thermal control of aerial optoelectronic platforms.

## 1. Introduction

As one of the information acquisition means, aerial optoelectronic platform has the advantages of flexibility, real-time, and detailed information. It plays an important role not only in civil surveying and mapping, but also in military applications, thus is the all around the world research focus. Since the temperature environment on the ground is quite different from the working environment in the air, changes in the external environment cause the aerial remote sensors to suffer from adverse conditions, resulting in inoperability or degraded performance.

The purpose of thermal control design is to determine which thermal control measures are taken in specific part of the aerial optoelectronic platform, how to allocate active thermal control resources, and how to control the internal and external heat exchange processes of the system, so that the temperature level and temperature gradient can meet the temperature index requirements in order to guarantee image quality. At present, many scholars have carried out a large number of theoretical studies and thermal equilibrium tests on the thermal analysis and thermal design of space optical systems [1,2,3,4,5,6,7,8], and the technology is relatively mature. However, there is not much research on the thermal control design of aerial optoelectronic platforms, and most of them focus on individual camera. Jongeun Choi et al. [9] presented the design and control of a thermal stabilizing system for an optomechanical uncooled infrared imaging camera. Yang Wengang et al. [10] proposed the thermal control index of a high-resolution space camera based on the wave phase difference distribution theory. On this basis, the thermal control technology was studied, and special thermal control measures were taken for the charged-coupled device (CCD). Fan Yue et al. [11] established the heat transfer model and thermal resistance network model of the lens and window components to optimize the heating power. Liu Weiyi et al. [12] studied the effects of temperature uniformity and temperature gradient on the imaging quality of aerial cameras, and an improved thermal control scheme was proposed based on the structural characteristics of the aerial camera, which effectively reduced the temperature gradient and improved the imaging quality. In addition, in Ref. [13], Liu Weiyi et al. focused on a thermal control strategy considering the contradiction between a low power consumption and high modulation transfer function (MTF) requirement. Liu Fuhe et al. [14] analyze the different impacts of temperature changes on the imaging resolution, and proposed an active thermal control method to improve the imaging quality of the sensor in a low-temperature environment.

Different from the previous thermal control research on aerial loads, the research object of this paper is not a single camera, but the whole optoelectronic platform, involving more components and more complicated thermal control methods are needed. In addition, due to the usage of a Cassegrain-reflecting objective lens, using active thermal control method with specific thermal control strategy to ensure the imaging quality of primary and secondary mirror is the focus. In this paper, on the basis of an aerial optoelectronic platform, the thermal control index was analyzed to ensure high-resolution image quality. Subsequently, the implementation of the active thermal control strategy and the combined thermal control scheme were elaborated. Next, thermal analysis was conducted under the extreme conditions by using thermal analysis software, and the effectiveness and correctness of the proposed thermal control technology were verified by simulations and experiments.

## 2. Analysis of Heat Flow Outside Working Environment of Aerial Optoelectronic Platform

Generally, the environment in which the aerial optoelectronic platform works is mainly divided into two situations: one is the ambient temperature range while parking on the ground, which is −40 °C~+60 °C; the other one is the air temperature (heat sink) range at aerial photography stage, which is −55 °C~0 °C. High-resolution camera thermal control index is very demanding which must be carefully analyzed for the entire heat flow environment outside the aerial optoelectronic platform. In fact, the external heat flow environment is complex and changeable, not only being affected by alternating direct sunlight, earth reflection, and infrared radiation of the earth, but also by factors such as different seasons, in and out of the earth shadow, and degradation of the coating. Here, the analysis of solar radiation, infrared radiation of the earth, and the external heat flow reflected by the earth to sunlight is illustrated as follows.

### 2.1. Solar Radiation 

The temperature of the sun is about 6000 K, and the energy it radiates into space is about 3.94 × 10^24^ W. 99.99% of the radiant energy is in the range of wavelength 0.18~40 μm, where the radiant energy of the band 0.15~0.3 μm corresponds to the radiation by black body of 4500 K while the radiation of the band 0.3~2.5 μm corresponds to that by the black body of 6000 K [15]. For the solar system planets, the earth-to-sun distance changes within a year frequently. The solar constants (the radiation density of the sun projected onto the earth) are shown in Table 1 for the spring equinox, summer solstice, autumnal, and winter solstice.

At an altitude of 8 km, as the atmosphere becomes thinner, the actual solar radiation intensity over time varies from 400 to 1376 W/m^2^. Generally, it is considered that there is no direct solar radiation at night.

### 2.2. Earth Reflection 

After solar radiation enters the earth atmosphere system, most of the energy is absorbed and reflected in the troposphere and the surface of the earth. The part that is reflected is called the Earth reflection, which includes the scattering of atmospheric molecules, the diffuse reflection of clouds, diffuse and specular reflections of soil, rocks, vegetation, waters, ice, and snow on the earth’s surface. According to the propagation characteristics of solar radiation in the atmosphere, Equation (1) is used to calculate the Earth reflection heat flow:

(1) $$q_{f}=\rho I_{0}\sin\theta$$

where ρ is reflectivity of the earth, the value of which is 0.20~0.46, $I_{0}$ is the total energy radiated by the sun to the earth, and θ is the angle of the earth reflection.

### 2.3. Infrared Radiation of Earth

When the earth receives radiation from the sun, it continually radiates heat to the space simultaneously. The solar radiation that falls on the earth is mostly absorbed by the earth and the atmosphere. The energy converted into heat is radiated in the form of long waves, that is, the infrared heat radiation of the earth. It is generally assumed that the spatial distribution of the infrared radiation of the earth is diffuse reflection, which can be equivalent to blackbody radiation. Therefore, ground infrared radiant heat flux density may be calculated by Equation (2)

(2) $$q_{IR}=\varepsilon \sigma T_{s}^{4}$$

where *ε* is the emissivity of the earth’s surface, *σ* is the Boltzmann constant, and T is the surface temperature of the earth. Different surfaces have different emissivity, and the surface temperature will fluctuate due to the change of day and night thermal environment. Considering the attenuation effect of atmospheric absorption when the surface infrared radiation is emitted into the atmosphere, the infrared radiation heat flux density at different heights is:

(3) $$q_{h}=\tau_{IR}q_{IR}$$

where $\tau_{IR}$ is the infrared radiation atmospheric transmittance, and $q_{IR}$ is ground infrared radiant heat flux density calculated by Equation (2).

Because it is difficult to determine the parameters involved in Equation (3), it is generally hard to calculate the earth’s thermal radiation accurately. The actual engineering problem could be calculated by the following equation roughly [16]:

(4) $$q_{k}=\left(1-\rho \right)S_{0}/4$$

where $S_{0}$ is e solar constant.

### 2.4. Convection

The convective heat flux density can be calculated using the following equation:

(5) $$q_{c}=h\cdot \Delta T$$

where, h is the convective heat transfer coefficient; ΔT is the temperature difference between the outer surface and the atmosphere. Before calculating the average surface heat transfer coefficient of the fluid sweep ball, Nusselt number, Nu needs to be calculated firstly using the following equation:

(6) $$Nu=2+\left(0.4Re^{\frac{1}{2}}+0.06Re^{\frac{2}{3}}\right)Pr^{0.4}{\left(\frac{\eta_{\infty }}{\eta_{w}}\right)}^{1/4}$$

where Nu is a dimensionless number, Re is Reynolds number, Pr is the Planck constant; and $\eta_{\infty }/\eta_{w}$ is the ratio of the aerodynamic viscosity to the aerodynamic viscosity at the spherical shell temperature. The speed of the optoelectronic platform with the aircraft is about 180 m/s, if the air temperature in the low temperature condition is −55 °C. After calculation, Nu is 1915.

According to the definition of Nu,

(7) Nu=hl/λ

h is the average convective heat transfer coefficient, l is the characteristic length, which is the diameter of the shell, and λ is the air thermal conductivity.

(8) h=(Nu·λ)/l

Finally, the convective heat transfer coefficient of the outer surface of the spherical shell is *h* = 62.9 W/(m^2^·°C).

Since the spherical shell has good sealing performance, it can be considered that the convection heat transfer inside the system is completed in the form of natural convection heat transfer. There are many internal components inside, and the shape is complex. It may take a lot of work to solve the convective heat transfer coefficient of each component. Thus, it is generally believed that the natural convective heat transfer coefficient is in the range of 2 W/(m^2^·°C) to 0 W/(m^2^·°C). Here, it is estimated that the conctive heat transfer coefficient between the internal components of the spherical shell and the air inside the sphere is 5 W/(m^2^·°C).

## 3. Thermal Control Design of Aerial Optoelectronic Platform

### 3.1. Structure of Aerial Optoelectronic Platform

**Figure 1:**
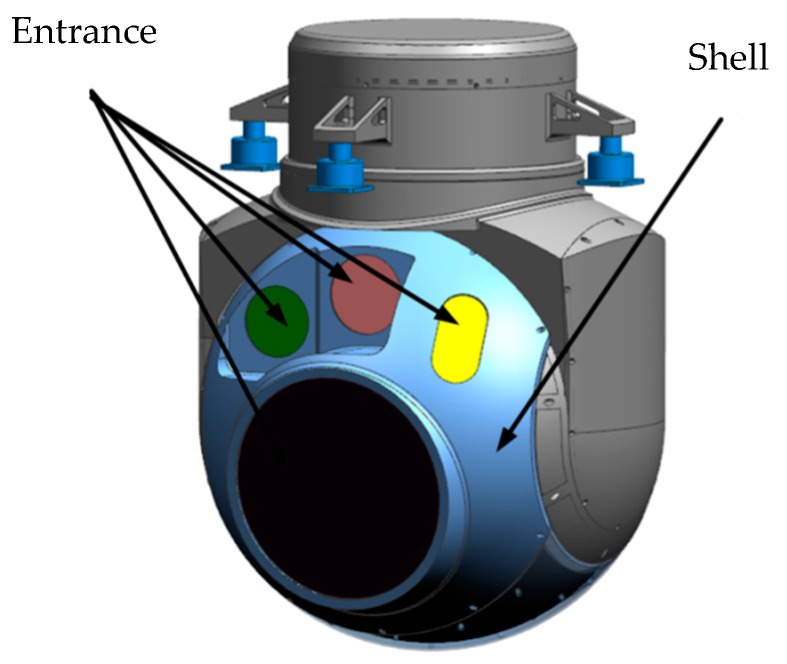
External structure of aerial optoelectronic platform.

**Figure 2:**
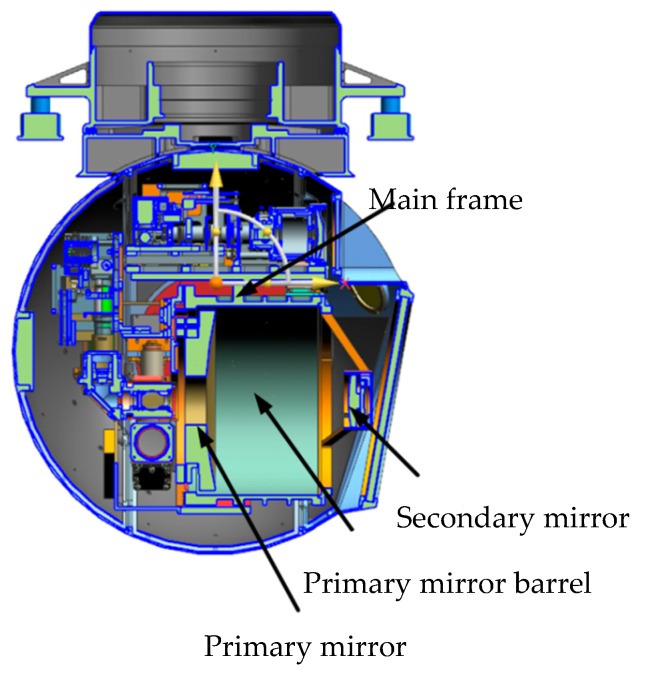
Optical components of aerial optoelectronic platform.

The entire optoelectronic platform system is 580 mm wide, 780 mm high, and weighs 110 kg. The structural material is aluminum alloy, and each component is electrically oxidized. The external structure of an aerial optoelectronic platform is shown in Figure 1 and the optical components are shown in Figure 2, including the primary mirror, the secondary mirror, the primary mirror barrel, and the main frame. The photoelectric loads share a Cassegrain-reflecting objective lens, where the main mirror is parabolic, and the secondary mirror is hyperboloid. The spectroscopic plate material on the inner side of the primary mirror is Crystallite, and a transmissive sub-image system is connected behind each imaging surface to realize long focal length, large aperture, and dual band imaging. The main frame is the structure that carries photoelectric loads, thus must have sufficient stiffness and strength.

### 3.2. Thermal Control Index

**Figure 3:**
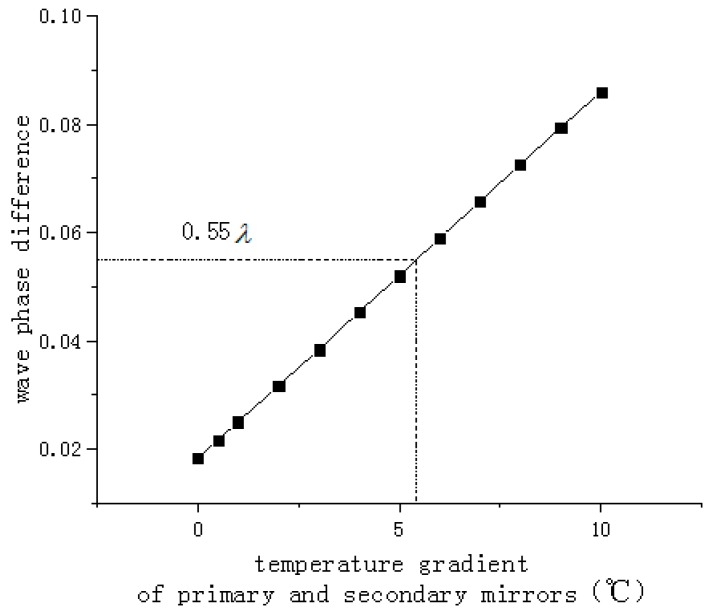
The influence curve of temperature gradient. Between primary and secondary mirrors on wave phase difference.

Aiming at the special working environment of the aerial optoelectronic platform, in view of the initial temperature uncertainty, the special characteristics of the rapid change of the working environment temperature and the thermal stability required for the long focal length loads, effective thermal control measures are needed. Liu Weiyi [12] analyzed the influence of temperature level and temperature gradient on the imaging ability of long focal length camera. The research showed that the system appeared large spherical aberration and coma when the temperature gradient changed. When the temperature level changed, the system had a large spherical aberration and relatively small coma. The effect of the spherical aberration may be partially compensated by the camera’s focusing function; however, the coma cannot be compensated. Therefore, it is considered that the effect of the temperature gradient on the image quality was higher than the temperature level. The wave phase difference is an index for judging the imaging quality of the optical system. The smaller the wave phase difference, the better the imaging quality of the system. In this paper, root mean square value of the system-wide wave difference assigned is not greater than 0.11*λ* in the optical camera design. The thermal tolerance is assigned to half of the total wave aberration, that is 0.055*λ*. Subsequently, through the thermo-optical analysis, the influence curve of temperature gradient between primary and secondary mirrors on wave phase difference is obtained, which is shown in Figure 3. When the wave phase difference is smaller than 0.055*λ*, the axial temperature gradient between the primary and secondary mirrors should be less than 5 °C. In other words, only an axial temperature gradient is less than 5 °C to meet the wave phase difference that the system can accept, thus ensuring the image quality. Generally, the aerial optoelectronic platform system’s working environment temperature ranges from −30 °C to 50 °C Increasing temperature of the loads helps to ensure a good working condition for the optical system. If the temperature of the working environment is too low, optomechanical structure may not work smoothly. At the same time, due to the limitation of the capacity of the heaters, taking into account the power that can be utilized by the thermal control unit, the temperature level of the primary mirror is set to −20 °C~+50 °C.

In summary, the thermal control index of an aerial optoelectronic platform system is set as:

(a) The temperature level of the primary mirror is expressed by the average temperature of three temperature measuring points $T_{avg}=\left(T_{ZJ1}+T_{ZJ2}+T_{ZJ3}\right)/3$ is within the range of −20 °C~+50 °C;
(b) The temperature gradient between the primary and secondary mirrors is lower than 5 °C.

Based on the analysis of the thermal control index requirements and the internal and external thermal environment of the system, an active-passive combined control technology is studied.

### 3.3. Passive Thermal Insulation Measures

#### 3.3.1. Covering Heat Insulation Layer

In order to reduce the influence of external environment changes on the temperature inside the system, the sensors and the shell are insulated. The heat insulating layer covering area is mainly divided into two parts: the inner surface of the spherical shell and the back surface of the secondary mirror heating cover (facing the secondary mirror).

**Figure 4:**
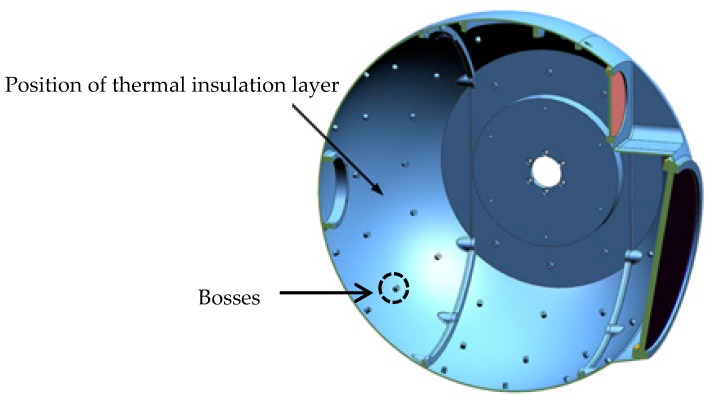
The position of the thermal insulation layer on the inner surface of the shell.

**Figure 5:**
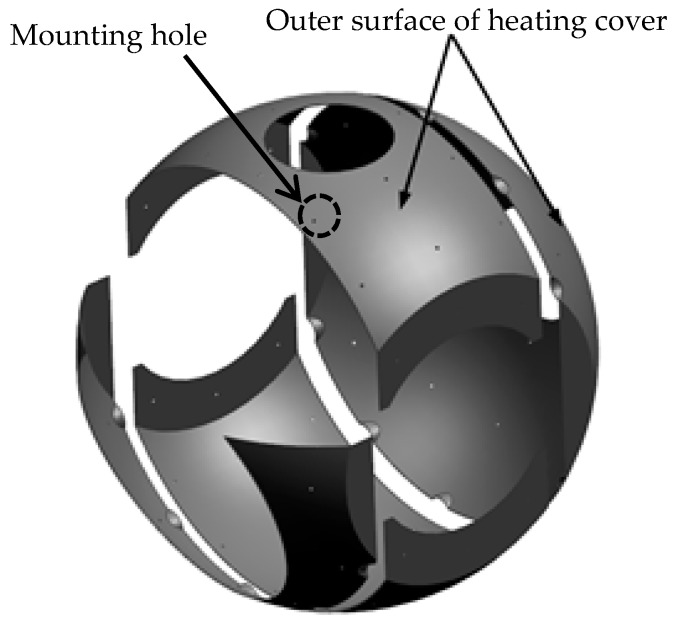
Insulation layer pasting surface on the heating cover.

The inner surface of the spherical shell is covered with a 6 mm polyurethane foam insulation layer (having good heat preservation performance, good waterproof performance, good bonding performance, and good aging resistance), except for the position of optical entrance and the position that inconvenient to be covered by the heat insulation layer. The insulation layer is attached to the inner side of the system with a double-sided aluminized polyester film, as shown in Figure 4. In order to achieve active thermal control, a heating cover with a thickness of 1 mm that has the same shape with the shell is added to facilitate the adhesion of the heating pieces, seen in Figure 5. It can be discovered in the figures that there is a plurality of 6 mm high cylindrical bosses on the shell with threaded holes. The shell heating cover has holes corresponding to the bosses. When installing, the shell heating cover is fixed by screws to the shell.

**Figure 6:**
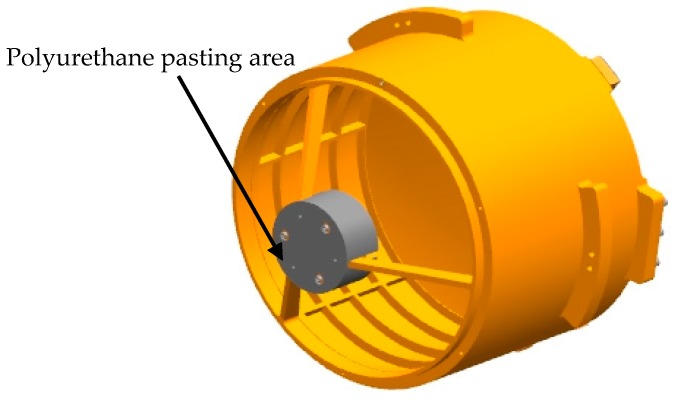
Insulation layer covering area on back of secondary mirror heating cover.

Similarly, in order to achieve active thermal control of the secondary mirror, a heating cover wrapped around the secondary mirror is designed. The back surface of the secondary mirror heating cover is covered with 6 mm thick polyurethane foam, and attached with a double-sided aluminized polyester film, as shown in Figure 6.

#### 3.3.2. Blackening

In order to ensure the uniform temperature of each component inside the system, blackening is performed at key parts. It is blackened by spraying black paint or black anodizing blackening treatment, and its emissivity is not less than 0.8. The blackened components are the main frame, optical element support components, the inner and outer surface of the primary mirror barrel and heating covers.

### 3.4. Active Thermal Control Measures

#### 3.4.1. Thermal Control Strategy

**Figure 7:**
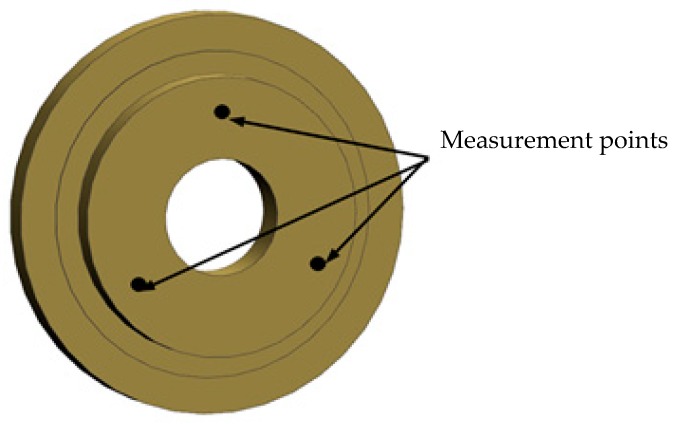
Measurement points on primary mirror.

The idea of active thermal control strategy is to measure the temperature of control points by temperature sensors, and then adjust the working state of the heater adhered on the heating cover, according to the temperature difference by a comparing with the target temperature, which makes the temperature of the control points reaches the target temperature. Here, the setting of the target temperature is divided into two cases depending on different areas: for the secondary mirror heating cover, in order to reduce the temperature gradient of the primary and secondary mirrors, the target temperature is set to the current average temperature of the control points on the primary mirror, as shown in Figure 7 (The primary mirror diameter is 260 mm, if only one point is used to represent the temperature level of the primary mirror itself, the radial temperature uniformity of the primary mirror cannot be taken into account. Considering the area of the primary mirror involved in this paper, three points on the radial center index circle within the range of the aperture diameter are selected, which are evenly distributed on the circumference. Taking the average temperature of these three points, the temperature of the primary mirror is characterized.); for other areas, the target temperature is the current average temperature of the primary mirror, but the target temperature range has to be controlled within −10 °C~30 °C. That means if the average temperature of the primary mirror is less than −10 °C, the target temperature is set to −10 °C. Similarly, if it is greater than 30 °C, the target temperature is set to 30 °C. This setting mode ensures the temperature inside the entire system is uniform and the working environment temperature for all loads more comfortable.

Switch type is adopted as temperature control mode for all the heating areas. The sensor used is DS18B20, a kind of digital sensor, which has the advantages of small size, strong anti-interference ability, and high precision. The heating piece is made of electric heating piece of 125 type polyimide film attached to the outer surface of the heating cover. The heating areas are controlled according to the switch type, of which control threshold is the target temperature ±0.1 °C. If the measure temperature is higher than the target temperature of 0.1 °C, the heating area is powered off. In contrast, if below the target temperature 0.1 °C, the heating area is heated. The control period of heating area is no more than 1 s.

#### 3.4.2. Implementation of Active Thermal Control Measures

**Figure 8:**
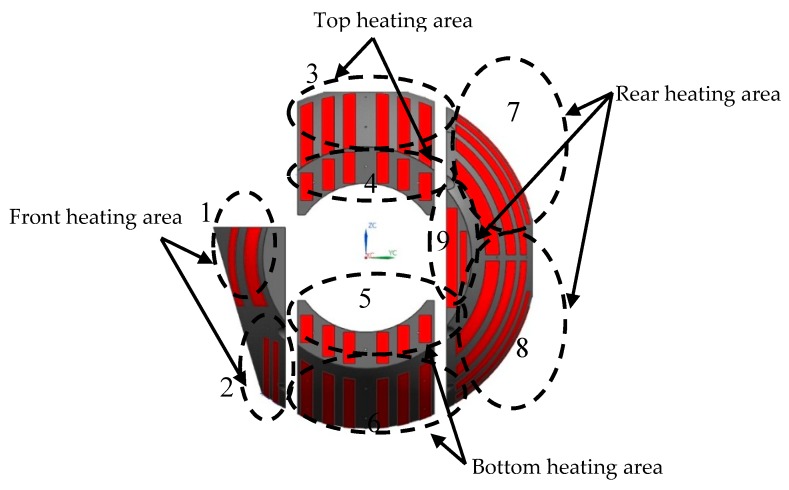
Heating areas on one side of shell heating cover.

**Figure 9:**
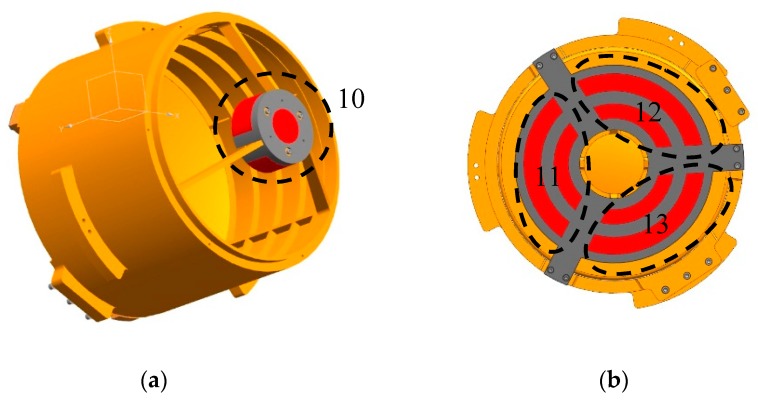
(**a**) Heating area of secondary mirror heating cover; (**b**) Heating area of primary mirror heating cover.

**Figure 10:**
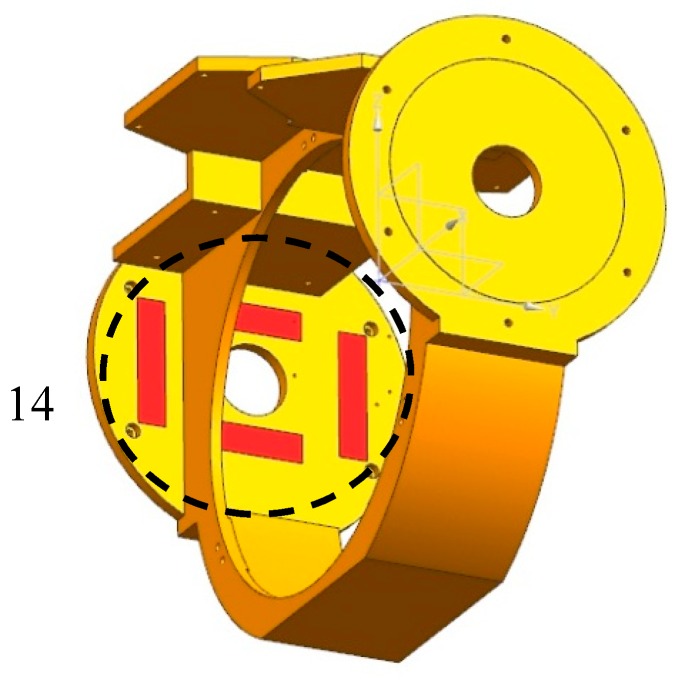
Heating area on one side of main frame.

In order to realize the thermal control strategy, heating cover is added. In addition to the shell heating cover and the secondary mirror heating cover mentioned above, the primary mirror heating cover is also designed on the back side of the primary mirror. The shell heating cover has a total of 18 heating areas, 4 on front, 4 on top, 4 on bottom and 6 on rear. The black dotted frames in Figure 8 denote heating areas on one side of the heating cover, and red strips denote the heating pieces. Heating pieces on the other side are symmetrically arranged. The purpose of this arrangement is to reduce heat leakage of the front and rear shell buckle lines. One heating area is provided on the back of the secondary mirror, as shown in Figure 9a, which may reduce the temperature gradient between the primary and secondary mirrors and increase the adaptability of the optical system to the environment. A total of three heating areas are provided on the primary mirror heating cover, as shown in Figure 9b, to ensure temperature uniformity. One heating area is arranged at each end of the main frame, where one-side heating area is shown in Figure 10. These two heating areas may reduce the leakage caused by strong convective heat transfer between the external low temperature environment and the shafts at both ends of the main frame. In summary, the entire system has 24 heating areas totally, and each heating area consists of a heating circuit. Thermal design power for different heating areas is shown in Table 2, where numbers in the first column denote heating areas in Figure 8, Figure 9 and Figure 10. It is worth to be noticed that the design power of symmetrical heating areas are equal.

## 4. Thermal Control Analysis and Results

**Figure 11:**
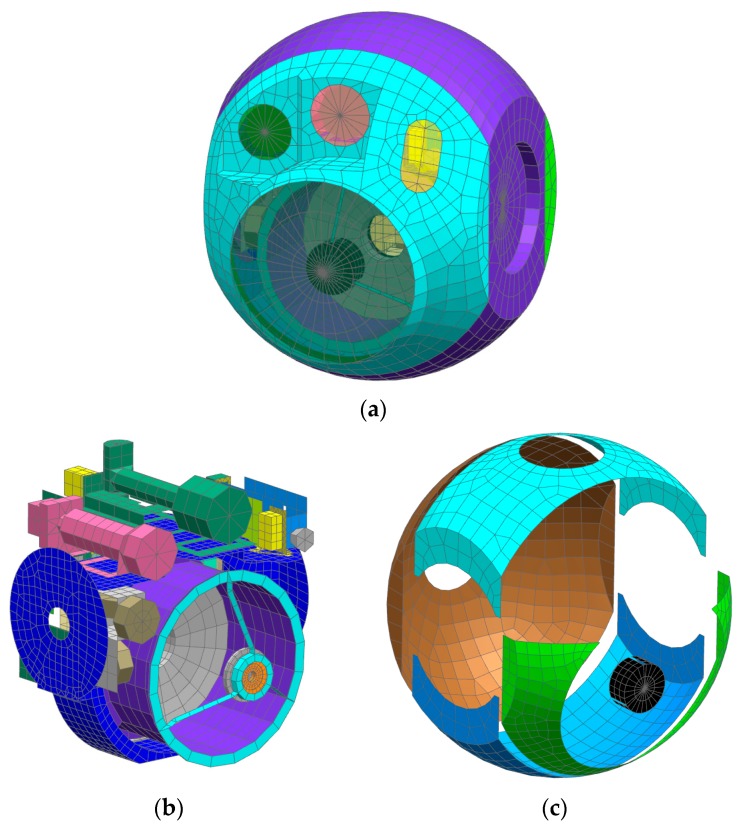
Thermal analysis model of aerial optoelectronic platform system. (**a**) outer grid structure (**b**) internal grid structure (**c**) shell heating cover grid structure.

Thermal analysis modeling and solving are done using the NX SST module. The thermal analysis model of the aerial optoelectronic platform system is shown in Figure 11. There are 9459 grid cells, 10,693 nodes, and 136 thermally coupled heat transfer channels being used in total. The main structural surface treatment and surface properties involved in the simulation process are shown in Table 3.

### 4.1. Thermal Analysis Conditions

According to the external heat flow, coating properties, sensor working mode, etc., the transient thermal analysis under extreme low temperature conditions is conducted, and the flight time is set at night, at which time the external heat flow has minimal influence, and the outer surface temperature of the shell is regarded to be equal to the ambient temperature in simulation. Combined with the heat flow analysis described in the second section, the specific operating conditions are shown in Table 4. The thermal analysis is conducted under the same operating conditions with active thermal control measure on and off, respectively.

### 4.2. Thermal Analysis Results

**Figure 12:**
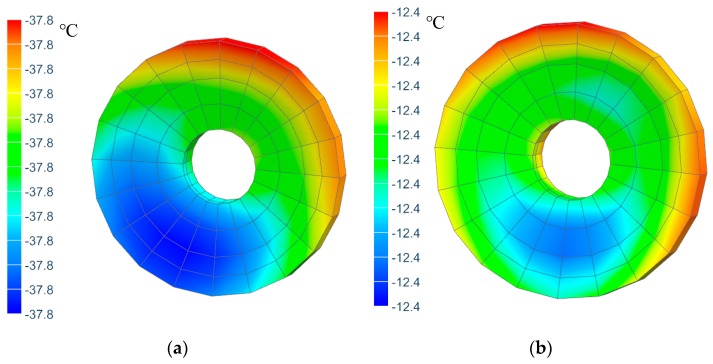
Primary mirror temperature distributions. (**a**) without active control; (**b**) with active control.

Figure 12 shows thermal analysis results of the primary mirror temperature under above mentioned operating condition with and without active thermal control measures respectively. It can be seen from the figure that when there is no active thermal control measures, the primary mirror temperature is lower than −30 °C, which doesn’t satisfy the temperature index that the temperature should be kept within −20 °C~+50 °C. In the case with active thermal control measures, the primary mirror temperature to −12.4 °C, meeting the temperature index requirements. Compared to uncontrolled conditions, the primary mirror temperature is increased by approximately 25 °C, making the primary mirror working environment more comfortable.

**Figure 13:**
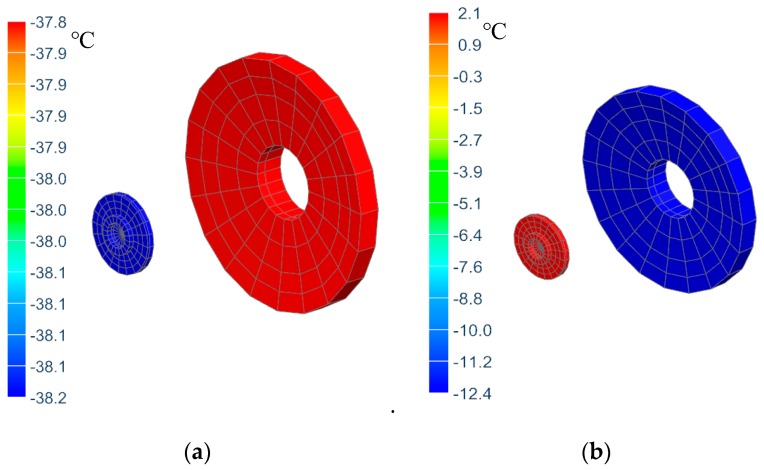
Temperature distribution of primary and secondary mirrors. (**a**) without active control; (**b**) with active control.

Figure 13 shows the temperature results of the primary and secondary mirrors. It can be found that the colors of the two mirrors are in the same range, respectively, indicating that the temperature uniformity is pretty good. In the case of no active thermal control, the temperature gradient of the primary and secondary mirrors is less than 2 °C. However, the temperature gradient is greater than 5 °C when there are active control measures. This is because it is unable to realize the temperature control strategy that the target temperature of the secondary mirror changes with the temperature of the primary mirror using the thermal analysis software (the thermal heating software sets the target temperature of the secondary mirror heating cover to a fixed temperature of 10 °C). In order to further assess whether the temperature gradient of the primary and secondary mirrors meets the thermal control index requirements with active thermal control measures, the test is necessary.

## 5. Thermal Control Test and Results

**Figure 14:**
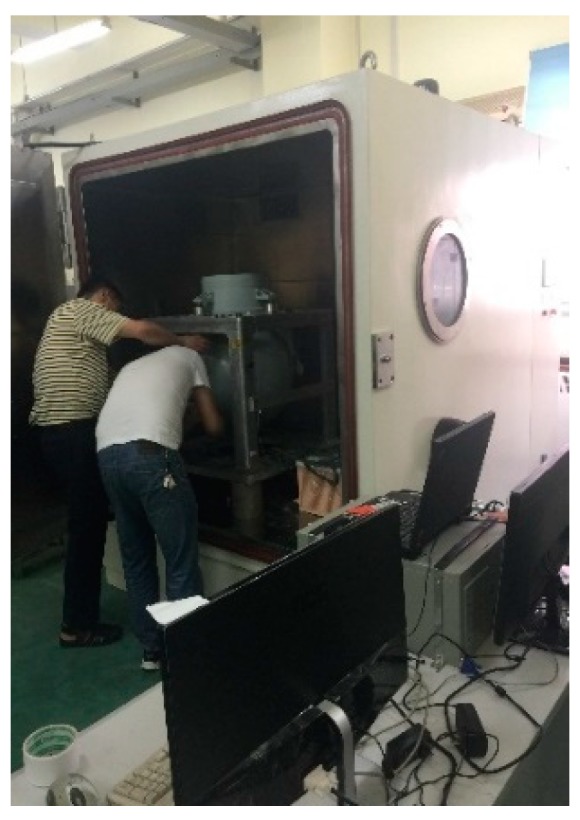
Test scenario.

In order to further verify whether the thermal control method proposed makes the temperature of the primary and secondary mirrors meet the index requirements, the tests are carried out under low temperature conditions with and without active thermal control measures, respectively. The atmosphere pressure is 30 kPa at the actual flight altitude of the aerial optoelectronic platform system. Due to the limitations of the test conditions, the pressure of chamber of high-low temperature and pressure cannot be reduced to 30 kPa. Considering that the sealing condition of the optoelectronic platform system is good enough, the test under normal pressure will enhance the influence of convective heat transfer and reduce the ability of the thermal control system to control the temperature through radiation heat transfer. Thus, the temperature control capability of the thermal control system under 30 kPa pressure conditions is better than that under normal pressure conditions. In other words, if the results from the test carried out under normal pressure conditions meet the temperature control index requirements, it can be considered that the test results at the pressure of 30 kPa must meet the index requirements as well. Figure 14 is the photograph of the test scenario.

### 5.1. Test Results

**Figure 15:**
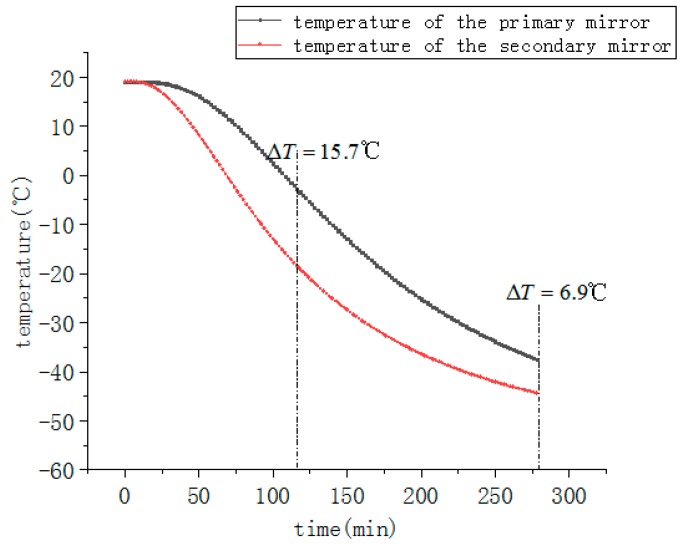
Temperature curves of primary and secondary mirrors. Without active thermal control measurements under normal conditions.

The temperature curves of the main measure points at low temperature test without active thermal control measurements are shown as Figure 15. Since the entire optoelectronic platform has no heat source, the temperature tends to decrease when the environment temperature is −55 °C. During the temperature drop process, there is a maximum temperature difference between the primary mirror and the secondary mirror. The temperature difference gradually decreases with time, and the whole system tends to be a low temperature steady state. Finally, the temperature of the primary mirror is stable at around −34 °C, which doesn’t meet the thermal control index requirements is the temperature level of the primary mirror should be between −20 °C~+50 °C. It can be determined from the curves that the maximum temperature gradient of the optical system is 15.7 °C. Although the temperature gradient decreases gradually over time, it is still higher than the 5 °C required by the thermal control index.

**Figure 16:**
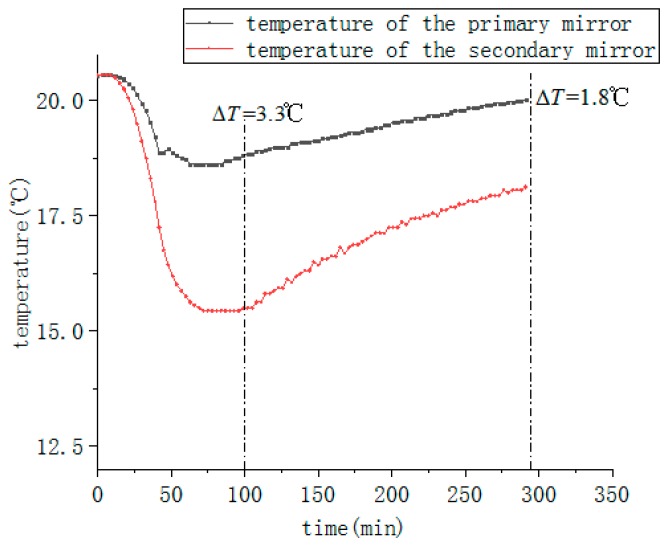
Temperature curves of primary and secondary mirror with active thermal control measurements under normal conditions.

Figure 16 shows the temperature curves of the main measure points at low temperature test with active thermal control measurements. It can be seen from the figure that after the active thermal control is turned on, although the environment temperature is at a low temperature of −55 °C, the temperature of the primary mirror is stable at about 20 °C, meeting thermal control index requirements. Further, the temperature gradient of the primary and secondary mirrors is less than 5 °C, which also meet the index requirements. And the range of temperature changes is significantly reduced, basically in equilibrium.

The test results are compared with the simulation results obtained in Section 4, as shown in Table 5. It is obvious that there is a certain gap between these two results. The reason is that the input conditions of the working conditions during the test and simulation are not completely consistent due to the limited test conditions, and the simulation software cannot realize the active control strategy accurately. The comparison results demonstrate that the verification of the proposed technology still relies mainly on the experimental means. Although the simulation analysis may obtain the temperature change trend qualitatively, and play an auxiliary role, it needs to be further improved in accuracy.

In combination with the simulation and test results, it is necessary to adopt active thermal control to ensure that the optical system meets the thermal control index requirements. Active thermal control makes the aerial optoelectronic platform system more adaptable to the effects of wide initial temperature and rapid changes of ambient temperature when working at different regions and different time periods.

### 5.2. Actual Imaging Quality

**Figure 17:**
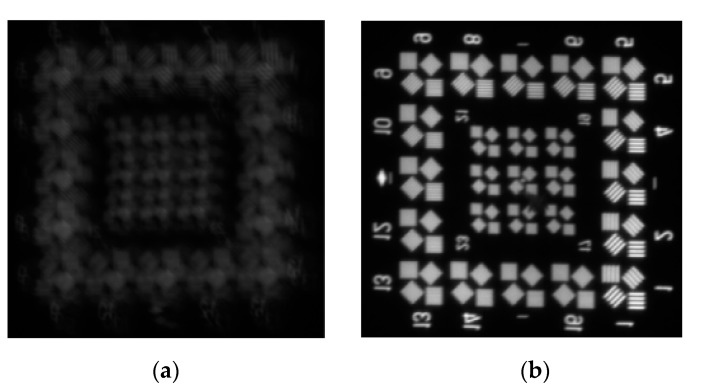
Imaging of the target under different conditions. (**a**) without active control; (**b**) with active control.

Figure 17 shows the images of the target in the simulated high altitude environment, without active thermal control and with active thermal control. The imaging quality is much better when there are active thermal control measurements, which shows active thermal control effective.

## 6. Conclusions

Based on the complex and variable working environment of the aerial optoelectronic platform, considering the structure itself, an active-passive combined thermal control scheme is proposed. Passive thermal control measures mainly use insulation materials. Based on effective control strategy, active thermal control measures utilize heating pieces adhered on the heating cover to achieve active electric heating control technology, especially local elaborate heating for the primary and secondary mirrors. After thermal analysis and experiment in extreme low temperature conditions, it is verified that the proposed combined method can improve the temperature level of the primary mirror, reduce the temperature gradient of the primary and secondary mirrors, and improve imaging quality effectively. It has certain guidance and references for the precision thermal control of aerial optoelectronic platform systems. In the future work, the active thermal control algorithm should be studied further to achieve precise thermal control.

## Figures and Tables

**Table 1:** Solar constant.

Time	Spring Equinox	Summer Solstice	Autumnal	Winter Solstice
Solar constant W/m^2^	1378	1323	1357	1412

**Table 2:** Thermal design power for different heating areas.

Nr.	Location	Thermal Design Power/W
1	Front heating area	10
2	Front heating area	14
3	Top heating area	16
4	Top heating area	40
5	Bottom heating area	20
6	Bottom heating area	40
7	Rear heating area	30
8	Rear heating area	30
9	Rear heating area	15
10	Secondary mirror heating cover	10
11	Primary mirror heating cover	5
12	Primary mirror heating cover	5
13	Primary mirror heating cover	5
14	Main frame	130

**Table 3:** Main structural surface treatment and surface properties.

Structural Components	Surface Treatment Status	Infrared Emissivity	Solar Absorption Rate
Inner surface of spherical shell	Paste double-sided aluminized polyester film	0.1	0.1
outer surface of heating cover	Paste double-sided aluminized polyester film	0.1	0.1
inner surface of heating cover	blacking	0.8	0.8
Primary mirror surface	coating	0.1	0.1
Secondary mirror surface	coating	0.1	0.1
Main frame	blacking	0.8	0.8
Main working loads	blacking	0.8	0.8

**Table 4:** Operating conditions.

Environment Conditions	Low Temperature without Active Thermal Control
Flight altitude (m)	8000
Atmospheric pressure (kPa)	30
Atmospheric density (kg/m^3^)	0.5
Solar radiation (W/m^2^)	0 Night flight
Infrared Radiation of the earth (W/m^2^)	270
Earth Reflection (W/m^2^)	0 Night flight
Initial temperature (°C)	−40
Environment temperature (°C)	−4~−55
Working duration	3 h

**Table 5:** Comparison of test results and simulation results.

Operation Condition	Low Temperature Without Active Thermal Control		Low Temperature with Active Thermal Control	
Method	test	simulation	test	simulation
Temperature of primary mirror (°C)	−34	−37.8	20	−12.4
Temperature gradient of primary and secondary mirrors (°C)	6.9	0.4	1.8	14.5
